# A pilot study to evaluate changes in pelvic floor muscle tone following pelvic organ prolapse surgery using a novel intra-vaginal pressure sensor device

**DOI:** 10.1007/s00192-022-05312-4

**Published:** 2022-08-08

**Authors:** James McConnell, Lynsey Murtagh, Molly Lim, Laura Pedofsky, Jennifer Kruger, David Budgett, Swati Jha

**Affiliations:** 1 Department of Urogynaecology, The Jessop Wing, Sheffield Teaching Hospitals, Tree Root Walk, Sheffield, S10 2SF UK; 2 Pelvic Floor Research Group, Auckland Bioengineering Institute, University of Auckland, Auckland, New Zealand

**Keywords:** Anterior colporrhaphy, Pelvic floor, Pelvic floor repair, Pelvic organ prolapse, Posterior colporrhaphy

## Abstract

**Introduction and hypothesis:**

Pelvic floor muscle weakness is a common cause of pelvic organ prolapse and urinary incontinence. Surgical repair of prolapse is commonly undertaken; however, the impact on pelvic floor muscle tone is unknown. The aim of this study was to compare the effect of anterior and posterior colporrhaphy on pelvic floor activation.

**Methods:**

Patients aged under 70 undergoing primary anterior or posterior colporrhaphy were recruited. Intra-vaginal pressure was measured at rest and during pelvic floor contraction using the Femfit® device (an intra-vaginal pressure sensor device [IVPSD]). Peak pressure and mean pressure over 3 s were measured in millimetres of mercury. The pre- and post-operative measurements were compared. The difference between the means was assessed using Cohen’s D test, with significance set at *p*<0.05

**Results:**

A total of 37 patients completed pre- and post-operative analysis, 25 in the anterior colporrhaphy group and 12 in the posterior colporrhaphy group. Anterior colporrhaphy showed no significant change in pelvic floor tone. Change in peak pressure was −1.71mmHg (−5.75 to 2.33; *p*=0.16) and change in mean pressure was −0.86 mmHg (−4.38 to 2.66; *p*=0.31). Posterior colporrhaphy showed a significant increase in peak pelvic floor muscle tone of 7.2 mmHg (0.82 to 13.58; *p*=0.005) and mean pressure of 4.19 mmHg (−0.09 to 8.47; *p*=0.016).

**Conclusions:**

Posterior colporrhaphy significantly improves pelvic floor muscle tone, whereas anterior colporrhaphy does not. Improved understanding of the impact of pelvic floor surgery may guide future management options for other pelvic floor disorders. Further work is needed to confirm the association of this improvement in pelvic floor disorders.

## Background

Twenty-five percent of women develop pelvic floor muscle weakness after childbirth, which can result in pelvic organ prolapse (POP) and/or urinary incontinence (UI). Twelve percent of these women will require surgery. The pelvic floor is a group of muscles and fascia that supports the internal pelvic organs in the correct anatomical position. These muscles are important for bladder and bowel control, along with sexual function [1]. Childbirth, ageing, obesity, chronic heavy lifting, and constipation can weaken the pelvic floor causing the pelvic organs to prolapse [2].

Pelvic organ prolapse may present as a dragging sensation, vaginal laxity or palpable vaginal bulge and causes a range of bladder and bowel symptoms. The Pelvic Organ Prolapse Quantification (POP-Q) system [3] produces a valid and reliable description of the site and severity of POP. On the other hand, pelvic floor muscle strength is assessed using the Modified Oxford Grading Scale (MOS) [4]; however, this assessment is subjective and varies between individuals.

Surgery for POP involves reinforcing the supporting fascia and repositioning the pelvic organs to restore their normal anatomical positions [1]. Surgery for POP often involves posterior colporrhaphy or anterior colporrhaphy. If there is prolapse of the cervix/uterus, a vaginal hysterectomy may be performed. However, the impact of surgery on pelvic floor activation/function, and intra-vaginal pressure, is poorly understood. There is also a significant risk of recurrence following surgery. There remains insufficient knowledge on the biomechanics of the pelvic floor to be able to accurately advise women on strategies to prevent POP recurrence after surgery.

A team at the Auckland Bioengineering Institute (ABI), Auckland University, have developed a novel intra-vaginal pressure sensor device (IVPSD; Femfit®) that measures pressure along the length of the vagina using eight sensors. The device was developed to measure pelvic floor muscle activation pressure and intra-abdominal pressure simultaneously, during a voluntary contraction, in order to assess the strength of the pelvic floor muscles. A proof-of-concept prototyping has already demonstrated that Femfit can provide reliable, objective measurements of a vaginal pressure profile and detect changes in pressure during pelvic floor contraction [5–7]. The reliability of the device has been demonstrated both within and between measurement sessions [8].

An objective assessment of changes in intravaginal pressure profiles pre- and post-surgery will provide a metric for assessing whether surgical intervention has improved the strength of the pelvic floor, or at least the ability of patients to perform a pelvic floor muscle contraction. This metric can be used objectively to assess the biomechanics of the pelvic floor, providing information on changes in anatomy and pelvic floor muscle strength following POP surgery and provide clinically valuable counselling pre-operatively.

This study is aimed at determining if there are changes in vaginal pressures before and after surgery for POP using Femfit, and whether posterior colporrhaphy has more of an impact on pelvic floor activation pressure than anterior colporrhaphy.

## Materials and methods

Patients were recruited prospectively from the urogynaecology clinic of a tertiary teaching hospital. The inclusion criteria required patients to be under 70 years of age, have a BMI of under 40 and for this to be primary prolapse surgery. Patients were excluded if they had had previous prolapse surgery, procidentia, vaginal infection or a contraindication to pelvic examination. Patients requiring both anterior and posterior compartment repair were also excluded. A sample size of 12 patients for each arm was calculated to provide sufficient power for this pilot study. Ethical approval for this study was obtained from the local research ethics committee (REC).

**Fig 1:**
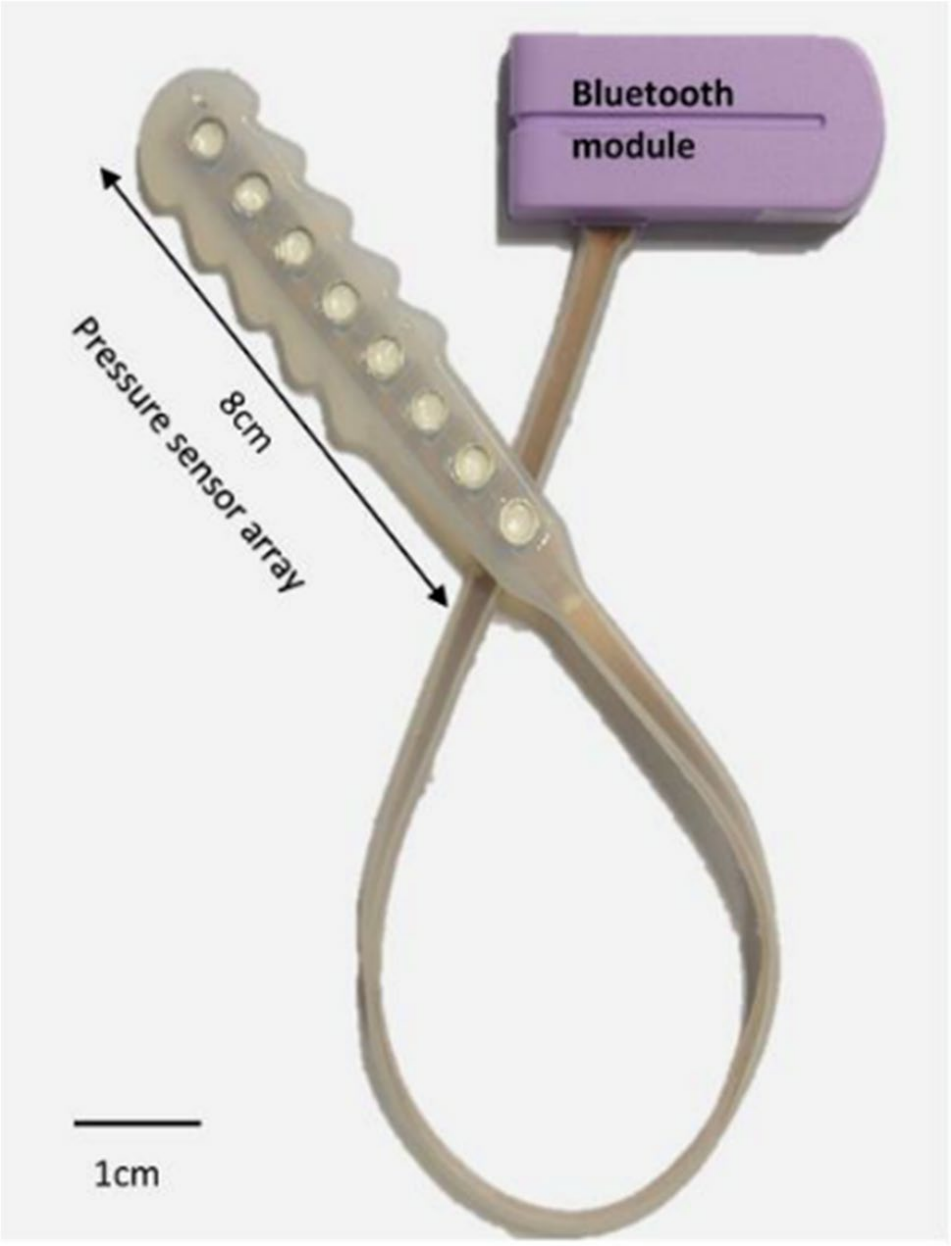
The intravaginal pressure sensor device

The Femfit® (Fig. 1) is a novel, wireless, pressure sensor array that conforms to the vaginal anatomy without introducing a pressure. The pressures exerted on the vaginal walls by pelvic floor muscles and connective tissues, including fascia, can be determined by the eight sensors, which are encased in a soft biocompatible silicone cover. Each sensor records pressure independent of the others, leading to eight pressure profiles along the length of the vagina. The deepest sensor lies above the pelvic floor and measures intra-abdominal pressure. As the position of the pelvic floor varies between patients, the three neighbouring sensors with the greatest increase in pressure from rest were used in the analysis, as these correspond to the pelvic floor muscle. This has been shown to be reliable and valid in previous studies [8, 9]. This enables the isolation of pelvic floor squeeze from intra-abdominal pressure and the effects of other tissues. Each sensor records pressure at a frequency of 140 Hz and transmits these data via Bluetooth to a custom-made portable Android tablet. The pressure data recorded on the table was analysed by the team in Auckland and then summary data were sent back to the clinical team.

At the pre-operative clinic patients were asked to complete the electronic Personal Assessment Questionnaire - Pelvic Floor (ePAQ-PF) and details were collected regarding their POP-Q score and their MOS. They then had baseline measurements taken with the IVPSD. While the patient was supine, intra-vaginal pressure measurements were obtained, with pelvic floor muscles at rest to be used as a baseline, during pelvic floor activation (3-s “squeeze” × three occasions) and during coughing (three occasions). The data from coughing were not used in the analysis as it was felt to be irrelevant to this study. Provided that Femfit was not expelled because of vaginal prolapse, these measurements were repeated with the patient standing.

All patients underwent surgery with three clinicians who use a standard technique of performing their anterior and posterior repair. Vaginal levator plication was not performed to avoid vaginal narrowing, as all women were sexually active.

Twelve weeks post-operatively participants returned to the clinic to complete the ePAQ-PF again to assess clinical change following surgery. Details of patients’ actual POP surgery, any post-operative complications, and their post-operative POP-Q and MOS scores were obtained from the clinical notes. Pelvic examination was also undertaken as part of routine clinical care and intra-vaginal pressure profiles were additionally recorded. The post-operative vaginal pressure data collection followed the pre-operative protocol. The pressure data from Femfit® were interpreted by the team in the ABI. They identified which sensors corresponded to the pelvic floor and extracted these data along with the intra-abdominal pressures. Demographic and clinical data were collated onto an Excel spreadsheet.

For each patient, the mean pressure exerted by the pelvic floor over 3 s and the mean peak pressure exerted were calculated pre- and post-operatively, both lying and standing. The results for both anterior and posterior colporrhaphy groups were collated to generate a mean peak pressure and 3-s mean pressure, pre-operatively and post-operatively, lying and standing. The difference between the means was assessed using Cohen’s D test with significance set at *p*<0.05.

## Results

A sample size calculation demonstrated that a sample size of 12 patients recruited to each arm would be sufficient to reject the null hypothesis in a pilot study. Sixty-three patients were recruited with 37 (58%) completing analysis. This included 12 in the posterior repair arm and 25 in the anterior repair. The discrepancy in group size was because recruitment continued until there were 12 completed patients in each group. Of those excluded: 8 patients had no post-operative data, 12 withdrew from the study, 1 patient had both anterior and posterior repair, 2 patients had incomplete data and 3 had device data collection issues. None of the patients withdrew because they found the device difficult to tolerate and feedback on the device was that it was very comfortable when fitted. There were no adverse reactions from the device.

Both groups were comparable in their parity, BMI, baseline MOS and grade of prolapse (Table 1).

**Table 1:** Demographics of the anterior and posterior repair groups

	Anterior repair	Posterior repair
Mean age (range)	59 (39–69)	54 (36–70)
Mean BMI (range)	27 (18–35)	30 (21–38)
Median parity (range)	2.3 (0–4)	2.5 (1–5)
Ethnicity	White 100%	White 100%
POP-Q—median (range)		
Aa	+1 (–2 to +3)	−2 (−3 to 0)
Ba	+1 (−2 to +3)	−2 (−3 to 0)
C	−1 (−6 to +3)	−5 (−6 to −2)
Ap	−2 (−2 to +1)	0 (−1.5 to +3)
Bp	−2 (−2 to +1)	0 (−1.5 to +3)
D	−4 (−6 to −2)	−7 (−7 to −5)
GH	4 (2-6)	5 (3-5)
PB	3 (2-3)	3 (1-3)
TVL	7 (7-10)	7 (6-10)
MOS mean (range)	1.4 (0-3)	1.5 (0-4)

*POP-Q* pelvic organ prolapse quantification score, *GH* genital hiatus, *PB* perineal body, *TVL* total vaginal length, *MOS* Modified Oxford Score

The results of the study are summarised in Tables 2, 3, 4, and 5. With the patient lying supine, in the anterior colporrhaphy group, the change in peak pressure was −1.71 mmHg (−5.75 to 2.33; *p*=0.16) and change in 3-s mean pressure was −0.86 mmHg (−4.38 to 2.66); *p*=0.31. In the posterior colporrhaphy there was a significant increase in peak pelvic floor muscle tone of 7.2 mmHg (0.82 to 13.58; *p*=0.005) and the change in 3-s mean pressure was 4.19 mmHg (−0.09 to 8.47; *p*=0.016). There was therefore a significant increase in peak pelvic floor muscle contraction and mean contraction strength over 3 s in the posterior repair group, but in the anterior repair group no significant difference was seen.

**Table 2:** Summary of results in the supine position: peak pelvic floor muscle tone

Type of colporrhaphy	Peak pelvic floor muscle tone (mmHg): mean (SD)		Difference (95% CI)
Anterior (*n*=25)	Pre-surgery	8.92 (8.54)	−1.71 (−5.75 to 2.33) *p*=0.16
	Post-surgery	7.21 (11.02)	
Posterior (*n*=12)	Pre-surgery	9.59 (10.04)	7.2 (0.82to 13.58) *p*=0.005
	Post-surgery	16.79 (11.02)

**Table 3:** Summary of results in the supine position: 3-s average pelvic floor muscle tone

Type of colporrhaphy	3-s average pelvic floor muscle tone (mmHg): mean (SD)		Difference (95% CI)
Anterior (*n*=25)	Pre-surgery	5.27 (7.44)	−0.86 (−4.38 to 2.66) *p*=0.31
	Post-surgery	4.41 (9.64)	
Posterior (*n*=12)	Pre-surgery	5.07 (6.73)	4.19 (−0.09 to 8.47) *p*=0.016
	Post-surgery	9.26 (7.79)

**Table 4:** Summary of results for the patient standing upright: peak pelvic floor muscle tone

Type of colporrhaphy	Peak pelvic floor muscle tone (mmHg): mean (SD)		Difference (95% CI)
Anterior (*n*=25)	Pre-surgery	4.14 (7.36)	1.63 (−1.92 to 5.18) *p*=0.36
	Post-surgery	5.77 (9.76)	
Posterior (*n*=12)	Pre-surgery	6.59 (5.67)	−0.17 (−3.77 to 3.42) *p*=0.96
	Post-surgery	6.42 (15.7)

**Table 5:** Summary of results for the patient standing upright: 3-s average pelvic floor muscle tone

Type of colporrhaphy	3-s average pelvic floor muscle tone (mmHg): mean (SD)		Difference (95% CI)
Anterior (*n*=25)	Pre-surgery	2.68 (5.81)	1.04 (−1.73 to 3.81) *p*=0.44
	Post-surgery	3.72 (7.59)	
Posterior (*n*=12)	Pre-surgery	3.73 (2.83)	−0.96 (−2.76 to 0.84) *p*=0.685
	Post-surgery	2.77 (8.45)	

With the patient standing there was no significant change in measured peak or mean pelvic floor contraction for either group.

## Discussion

To our knowledge, this is the first study of its kind and the first use of the Femfit® in clinical practice. As a pilot study we found a consistent effect within groups and patients found the device and the protocol to be acceptable. We had a diverse group of patients in terms of age, BMI and parity. All of our patients were white, although this is unlikely to have affected the results.

Although the fascial plication could have played a contributory mechanism, we do not feel that this was the main factor influencing the outcomes. Both anterior and posterior vaginal repairs involved fascial plication, but improvements were seen in only posterior repairs; hence, this explanation does not follow.

We were limited by a high rate of patient withdrawal during the study, with 42% of those recruited not completing the analysis. This particularly affected the posterior repair arm with 15 out of 27 patients (56%) not completing the study. It is unclear why there was such a high withdrawal rate from the posterior repair arm but this will not have affected the statistical power of the results. As the study population consisted only of white women, it is difficult to extrapolate the results to other ethnic groups. In addition, all measurements were done in the early post-operative period; thus, it is difficult to know whether these improvements were sustained over time or whether they translated into better pelvic floor function with reduced urinary incontinence and/or improved sexual function, as patients had not resumed routine exercise or intercourse when they were seen post-operatively.

This is the first study of its kind to assess measurement of pelvic floor muscle contraction before and after pelvic floor repair, to the best of our knowledge. It is unclear why there would be an effect seen with the patients supine but not standing. A study by Morgan et al. [10] found that intravaginal closing force is significantly higher when standing than when lying. They suggest that, when standing, there is a significant increase in intra-abdominal pressure and resistance within the pelvic floor muscles that serves to close the vagina. This increase in intra-abdominal pressure and the alteration in basal tone of the pelvic floor when standing may have reduced the effect of conscious squeeze when the patient is standing. This may also suggest that factors such as BMI might play a significant role in the efficacy of pelvic floor tone, as abdominal pressure is significantly affected by obesity and increasing weight. This highlights the importance of weight loss for effective pelvic floor muscle training.

It is also possible that patients are better able to perform pelvic floor squeeze when lying supine, as they are able to isolate the movement with the rest of the abdominal and pelvic muscles relaxed. A previous study found that despite a significant increase in intravaginal pressure when stood there was no difference between the pelvic floor muscle squeeze when standing and that when lying supine [11]. In that study they used a balloon pressure sensor, which would not have the benefit of measuring the length of the vagina or definitely aligning with the pelvic floor, and that may explain why our results differed, as we could isolate the pelvic floor contraction from the range of sensors and also confidently remove the intra-abdominal pressure effect.

The hypothesis was that posterior colporrhaphy would increase pelvic floor muscle contraction strength because the nature of the surgery may draw together and reinforce component fibres of the pelvic floor. This seems to have been the case when the women were lying supine, although not when they were standing. The implications of this are unclear and further work is needed to understand the impact of this change on the functioning of the pelvic floor and the symptoms that patients may experience as a result of pelvic floor dysfunction (e.g. urinary incontinence). This device may be beneficial when teaching women pelvic floor exercises as they can get real-time feedback on their pelvic floor contraction and it allows their care provider to see how they are doing as well. There is an added benefit that this device is more reliable than subjective scoring, as it is an objective measure of pelvic floor contraction.

Areas for future research include work to better understand the impact of pelvic floor activation pressure on symptoms associated with pelvic floor dysfunction and whether the changes seen here have a clinical implication. We are also using the Femfit® to study changes in pelvic floor tone during and after pregnancy to understand the impact of pregnancy on the pelvic floor and to better understand the underlying pathophysiology of postpartum pelvic floor dysfunction.
